# Long-term intraocular pressure fluctuation in patients with stable glaucoma: the impact of regression to the mean on glaucoma management

**DOI:** 10.5935/0004-2749.202100116

**Published:** 2021

**Authors:** Gustavo Henrique de Lima Melillo, Ana Luiza Bassoli Scoralick, Fábio Nishimura Kanadani, Carolina Pelegrini Barbosa Gracitelli, Augusto Paranhos Jr, Tiago S. Prata

**Affiliations:** 1 Department of Glaucoma, Instituto de Olhos Ciências Médicas, Belo Horizonte, MG, Brazil; 2 Department of Ophthalmology, Escola Paulista de Medicina, Universidade Federal de São Paulo, São Paulo, SP, Brazil; 3 Department of Ophthalmology, Mayo Clinic, Jacksonville, FL, USA; 4 Centro de Estudos Alcides Hirai, Ver Mais Oftalmologia, Vinhedo, São Paulo, SP, Brazil

Dear Editor,

Despite the growing awareness regarding the risk factors associated with the development and progression of glaucoma, intraocular pressure (IOP) remains the only modifiable risk factor; however, the method for managing it in routine clinical practice remains debatable^(1)^. The role of IOP fluctuations as a risk factor for glaucomatous progression is controversial and depends on attributes such as the damage level^(2,3)^. Moreover, there is no consensus on the optimum approach to analyze the IOP variation, and clinical decisions often lack evidential support. In this regard, clinicians must consider that IOP constantly changes because of several factors that determine the existence of daily and inter-visit fluctuations^(4)^. Considering these inter-visit fluctuations, it should be noted that statistical phenomena, such as regression to the mean, could influence the clinician’s perception of IOP to change over time^(5)^; moreover, the following question arises: what would be an acceptable IOP fluctuations or peak for treated stable glaucoma patients in routine clinical practice?

To achieve a deeper understanding of IOP behavior between visits in patients treated for glaucoma, we reviewed all charts of consecutive patients with stable open-angle glaucoma (OAG) to determine their long-term IOP profile over 5 years. The included patients had neither anatomical nor functional evidence of progression (measured using disc photos/retinography and reliable visual field tests) within the inspected interval, and no changes were made in the medical regimen during the follow-up period. Eyes with previous laser or filtering glaucoma surgery were excluded. All IOP measurements were performed, and for each patient, we calculated the mean long-term and peak IOP values. The IOP measurement in the first subsequent visit after the peak was also recorded (post-peak IOP). The following were the major outcome measures: (1) analyses of the IOP distribution values, based on central tendency (mean and median) and dispersion metrics (standard deviation and percentiles) and (2) comparison between the post-peak IOP measurements and the mean IOP values (paired t-test). The study was conducted according to the principles in the Helsinki Declaration.

**Figure 1:**
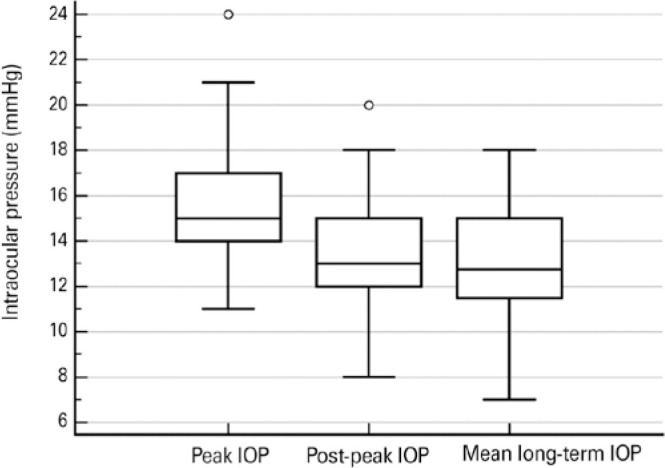
Box-and-whisker plots showing the distribution of intraocular pressure (IOP) values at the following three different time points: peak IOP (maximum value during follow-up), post-peak IOP (IOP in the following visit after the peak was documented), and mean long-term IOP. While the central box represents the median and interquartile range, the whiskers depict the minimum and maximum values. Outlier measurements are displayed as separate dots.

Thirty stable OAG patients (30 eyes) with a mean age of 64.4 ± 12.9 y were included in the analyses. Patients had a mean visual field deviation index of -3.9 ± 4.3 dB and used a median of 2 (interquartile range; 1-2) glaucoma medications. The mean and peak IOP values were 13.3 ± 2.6 mmHg and 16.1 ± 2.9 mmHg, respectively. Overall, the patients had a mean-positive IOP variation of 2.8 ± 1.3 mmHg above their mean long-term values. In fact, based on the 95^th^ percentile, only 5% of the patients presented elevations of >4.5 mmHg above their mean long-term IOP. More interestingly, we observed that after the IOP peak was documented, IOP revealed a significant tendency to regress toward the average at the subsequent visit (without any changes in the existing medical regimen) because there was no significant difference between the post-peak IOP (13.4 ± 2.8 mmHg) and long-term mean IOP values (p=0.736; Figure 1). In fact, in one-third of the eyes, the regression surpassed the mean value. Particularly, the post-peak IOP was lower, equal, or higher than the long-term mean in 33.3%, 30%, and 36.6% of the cases, respectively.

Although our data were derived from a relatively small sample of treated OAG patients, our data are based on a specific and unique population that was followed up for at least 5 years, had stable disease, had an unchanged medical regimen, had never undergone glaucoma surgery, and were adequately selected to answer the main study questions. Our findings suggest that positive IOP variations (up to 4.5 mmHg in 95% of the cases) can occur even in stable OAG eyes, and this increase does not necessarily trigger a sustained rise. Therefore, therapy escalation based on a single IOP peak is an unwarranted approach, and a sustained rise (or disease progression) must be confirmed before treatment enhancement. Moreover, whenever a treatment change is considered necessary after a significant IOP rise, it should be noted that the physician’s perception on medication effectiveness may be influenced by regression to the mean because the post-peak IOP tends to decrease toward the average in most cases. We believe that this may prevent overtreatment and its impact on individuals and health systems.
